# Moso bamboo (*Phyllostachys edulis* (Carrière) J. Houzeau) invasion affects soil microbial communities in adjacent planted forests in the Lijiang River basin, China

**DOI:** 10.3389/fmicb.2023.1111498

**Published:** 2023-02-21

**Authors:** Hongping Sun, Wenyu Hu, Yuxin Dai, Lin Ai, Min Wu, Jing Hu, Zhen Zuo, Mengyao Li, Hao Yang, Jiangming Ma

**Affiliations:** ^1^Key Laboratory of Ecology of Rare and Endangered Species and Environmental Protection (Guangxi Normal University), Ministry of Education - Guangxi Key Laboratory of Landscape Resources Conservation and Sustainable Utilization in Lijiang River Basin, Guilin, China; ^2^College of Life Science, Guangxi Normal University, Guilin, China; ^3^College of Biology and Pharmacy, Yulin Normal University, Yulin, China

**Keywords:** moso bamboo invasion, karst soil, microbial community, Lijiang River basin, planted forests

## Abstract

**Introduction:**

Moso bamboo (*Phyllostachys edulis* (Carrière) J. Houz.), the most widely distributed economic bamboo species in southern China, can easily invade adjacent communities due to its clonal reproduction. However, there is little information on the effects of its establishment and expansion to adjacent forest soil communities, particularly in planted forests.

**Methods:**

We investigated the relationships between soil properties and the microbial community during bamboo invasion under different slope directions (shady or sunny slope) and positions (bottom, middle, or top slope), in three typical stand types (bottom: pure moso bamboo, middle: mixed stands of moso bamboo and Masson pine (*Pinus massoniana* Lamb.), and top: pure Masson pine) in the Lijiang River Basin. This study aimed to explore the effects of key environmental factors on soil microbial composition, diversity, and abundance.

**Results and Discussion:**

The results showed that the abundance of *Acidobacteria* bacterium and *Acidobacteria* bacterium 13_2_20CM_58_27, and *Verrucomicrobia* bacterium decreased as the slope increased (*p* < 0.05), whereas the abundance of *Alphaproteobacteria* bacterium, *Actinobacteria* bacterium, *Trebonia kvetii*, and *Bradyrhizobium erythrophlei* increased as the slope increased (*p* < 0.05). However, the difference of slope direction on microbial communities was not significant. The pH, organic matter (OM) and total phosphorus (TP) were the key soil environmental factors; most microorganisms (*Betaproteobacteria* bacterium, *Candidatus Eisenbacteria* bacterium, *Betaproteobacteria* bacterium SCGC_AG − 212 − J23, *Gemmatimonadetes* bacterium, *Actinobacteria* bacterium 13_2_20CM_2_66_6, and *Myxococcaceae* bacterium) showed a positive relationship with pH and a negative relationship with OM and TP. Slope position significantly affected OM, calcium (Ca), total nitrogen (TN), available phosphorus (AP), hydrolyzed nitrogen (HN), pH, and microbial abundance and composition. Slope direction significantly affected TP and magnesium (Mg). The structural equations also indicated that slope position had an effect on microbial composition, abundance, and diversity. Slope position was negatively correlated with pH (*r =* −0.333, *p* = 0.034) and positively correlated with OM (*r =* 0.728, *p* < 0.001), TN (*r =* 0.538, *p* < 0.001) and Ca (*r =* 0.672, *p* < 0.001); pH was positively correlated with microbial composition (*r =* 0.634, *p* < 0.001), abundance (*r =* 0.553, *p* < 0.001) and diversity (*r =* 0.412, *p* = 0.002), TN was positively correlated with microbial composition (*r =* 0.220, *p* = 0.014) and abundance (*r =* 0.206, *p* = 0.013), and Ca was negatively correlated with microbial composition (*r =* −0.358, *p* = 0.003) and abundance (*r =* −0.317, *p* = 0.003). Slope position can also influence microbial composition (*r =* 0.452, *p* < 0.001) directly. In addition, slope direction had an indirect effect on microbial diversity through total potassium (TK). Therefore, we proposed that the different variations in microbial community during bamboo invasion could be related to the influence of invasion on the soil properties at different invasion stages.

## 1. Introduction

Moso bamboo (*Phyllostachys edulis* (Carrière) J. Houz.) is a widely planted herb in the forest area in southern China due to its economic value (Ouyang et al., 2022). According to the eighth national forest inventory, the existing bamboo canopy in China covers an area of about 6.01 × 10^6^ km^2^, of which moso bamboo canopy covers about 4.43 × 10^6^ km^2^ (Xu et al., 2020). Moso bamboo is able to expand rapidly because of its strong underground rhizome, which can quickly extend and invade neighboring communities, producing bamboo shoots. This enables clonal expansion of populations into new habitats and increases the area of bamboo forests, gradually leading to the replacement of existing vegetation with moso bamboo. The worldwide distribution of moso bamboo is rapidly expanding (Li et al., 2017). Previous studies have found that the invasion of moso bamboo has caused reductions in plant biodiversity (Ouyang et al., 2016), productivity (Song et al., 2017), and litter production (Song et al., 2016). In the Tianmu Mountain Nature Reserve, moso bamboo invasion caused substantial changes in plant species diversity and had strong negative effects on plant communities (Song et al., 2013). Tian et al. (2020) found that moso bamboo invasion decreased element concentrations in litter and soil as well as total microbial abundance and diversity in subtropical forests in southern China. Masson pine (*Pinus massoniana* Lamb.) forests have been planted for a long time in southern China (Chen et al., 2016). Currently, many Masson pine forests in the Lijiang River Basin have been invaded by moso bamboo, significantly affecting soil properties (Guan et al., 2017). These forests have faced many problems, including low species diversity, simplified structure (monoculture with a poorly developed shrub and herb layer), and poor resistance to natural disturbances. However, few studies have examined the effects of moso bamboo invasion on soil microbial communities of Masson pine, it is also unclear how microbial abundance, composition, and diversity respond to multiple disturbances, or whether multiple environmental changes lead to unanticipated interactive effects (Shang et al., 2021). Consequently, it is urgent to study the impact of moso bamboo invasion on Masson pine forests for ecological stability of the Lijiang River Basin.

Recently, moso bamboo invasion has been a common phenomenon in natural secondary broadleaf, and coniferous forests in the south of China (Xu et al., 2020). In addition, moso bamboo invasion can cause changes to the original soil properties. For example, Ma et al. (2022) studied the impact of moso bamboo invasion on evergreen broad-leaved forests in Anji County, Zhejiang Province, and found that the invasion of moso bamboo increased soil pH and decreased soil organic matter (OM) content. Previous studies (Fukushima et al., 2015; Shiau and Chiu, 2017) have reported the effect of moso bamboo invasion on soil properties, such as soil labile carbon (C) and nitrogen (N) contents. Liu et al. (2019) also found similar results when studying moso bamboo invasion into coniferous forests, which would lead to the decrease of soil C and N content, but had no significant difference on soil phosphorus (P) content. However, Li et al. (2017) indicated that the pH and total nitrogen (TN) levels of the soil in mixed forests during moso bamboo invasion were higher than those in uninvaded and bamboo forest soils. The invasion of adjacent hinoki (*Chamaecyparis obtusa*) forests by moso bamboo lead to an increase in soil pH, resulting in significant and sensitive changes in soil exchangeable calcium contents (Umemura and Takenaka, 2015). Plant invasions can seriously affect soil properties such as soil moisture, pH, and effectiveness of soil nutrients (N and P) (Zhang et al., 2019).

Soil microbial communities are an important component of forest ecosystems, can respond to environmental changes and are remarkably influenced by soil properties (Shang et al., 2021). Waymouth et al. (2020) indicated that microbial communities are closely related to soil properties, such as pH, total phosphorus (TP), and TN. Previous studies indicate that pH is a key factor in directly and indirectly determining organic phosphorus-mineralizing-related gene abundance, which in turn affects microbial diversity (Wan et al., 2021). Soil pH has substantial effects on soil fungi, actinomycetes, and other microorganisms, the abundance of which decreases with increasing soil pH (Shangguan et al., 2019). Widdig et al. (2020) found that TN affected microbial composition by adding TN and TP to a grassland soil. Therefore, plant invasion may impact soil microbial community by influencing soil properties and may have different effects on the abundance, composition, and diversity of the microorganisms (Shangguan et al., 2019; Widdig et al., 2020; Wan et al., 2021).

Karst surface is composed of carbonate minerals and the soil layer is shallow, easily eroded, and degenerative. Owing to the unique geographical location and topography of the Guilin National Forest Park, a sequence of various stages of moso bamboo invasion can be observed on the bottom, middle, and top slopes. This provides unique conditions to study changes in soil properties and the structure of microbial communities. The objective of the present study was to address the following questions: (1) What are the variations in soil microorganism characteristics at different invasion stages? (2) What are the key soil environmental factors that affect the microbial community? and (3) How do the slope direction and position influence microbial communities? This study focused on the impact of moso bamboo invasion on soil microorganisms in the Lijiang River Basin. Studying the response in soil microbial community composition and diversity to variations in soil properties in different expansion stages will elucidate the key factors involved in plant invasion, thus providing scientific guidance for the restoration and sustainable management of forest vegetation.

## 2. Materials and methods

### 2.1. Sample collection and measurement

**Figure 1:**
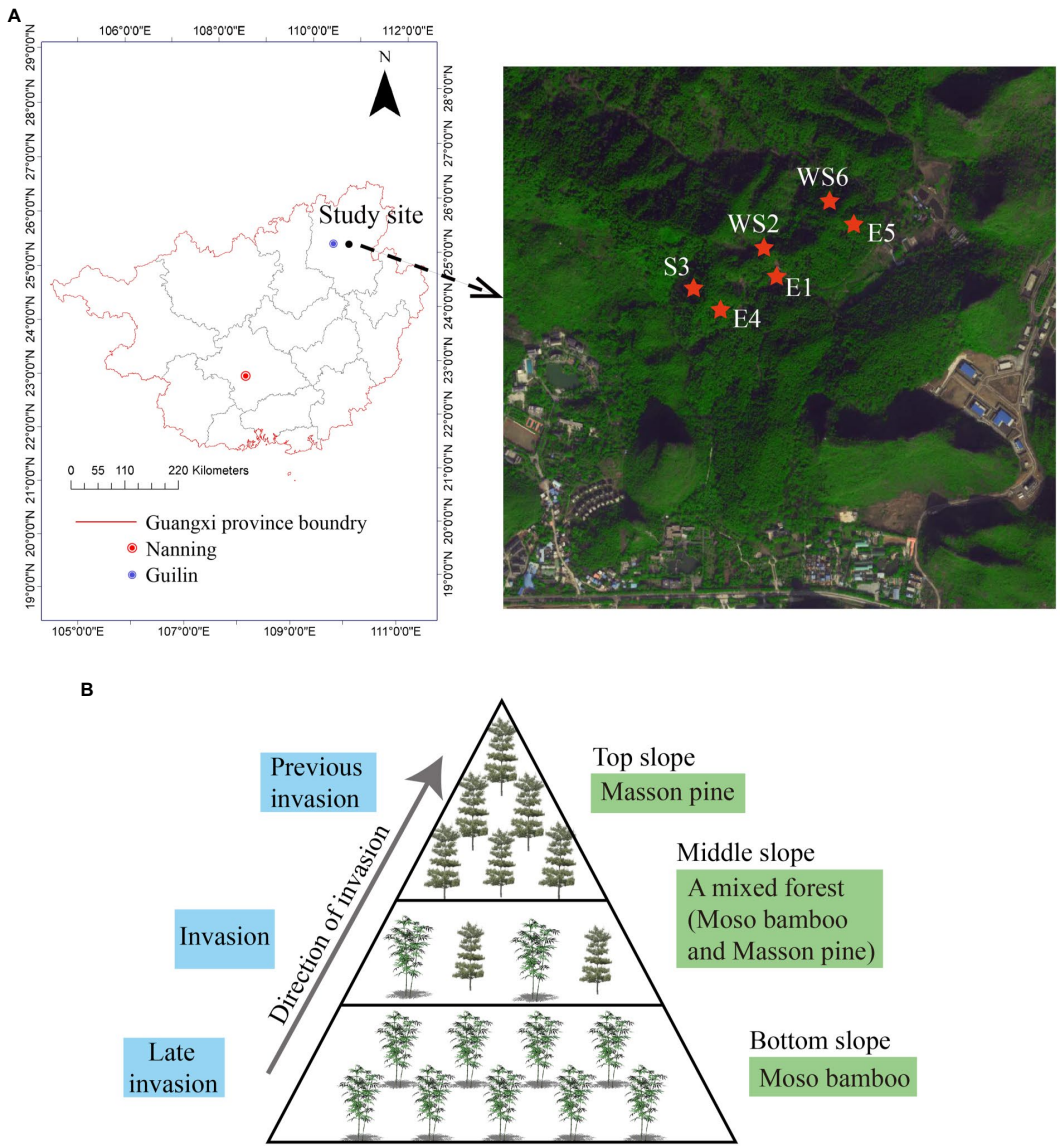
Study area settings of different slope directions **(A)** and different slope positions **(B)** in Guilin National Forest Park.

The study was conducted in the Guilin National Forest Park (25°13′31.46″ N, 110°14′51.37″E), Guangxi Province (Figure 1A). The park is located in the Lijiang River basin area of southwestern China, which is a typical karst landscape. The park has a total area of 581.5 hm^2^ and a forest area of 318.1 hm^2^. The climate is humid, the annual average temperature is 19.2°C, and the annual average precipitation is 1930.6 mm. The soil type is principally laterite and yellow soil based on the Chinese soil classification; this soil texture is classified as Xanthic Ferralsols in the World Reference Base for Soil Resources (Xu et al., 2020). The soil is generally thin with a coarse texture. The Masson pine forest in the park has a planting history of more than 7 years. After long-term forest closure, it has been transformed into a low-mountain bamboo experimental forest as its care measures, and the management measures are unified. We chose moso bamboo for this investigation on the basis of long-term and continuous research, because the average age of the bamboo is 5 years. Moso bamboo characteristically grows in a 2-year cycle, after 3–5 years of growth, the plant is more vigorous (Lin et al., 2022). Thus, the phenomenon of moso bamboo invading the Masson pine forest occurs at the fifth year.

Sampling of a moso bamboo invasion, in Guilin National Forest Park, was conducted on sunny and shady slopes at the bottom, middle, and top areas of a representative mountain system; sample collection was repeated three times in the same mountain. The different stages of the moso bamboo invasion could be observed on the bottom, middle, and top slopes of Guilin National Forest Park. The bottom slope was covered with moso bamboo; the middle slope was a mixed forest of moso bamboo and Masson pine; the top slope was covered with Masson pine (Figure 1B). This provides a unique condition for studying the changes of soil properties and microbial community structure. We simulated three invasion states of bamboo based on these three stand states. The top slope (T) was considered the previous invasion period, the middle slope (M) was considered the invasion period, and the bottom slope (B) was considered the late invasion period. Natural forest was used as a control group (CK). The shady and sunny slopes of the mountains are, respectively, numbered as E1, E4, and E5 with WS2, S3, and WS6, respectively (Pan et al., 2022). Therefore, the code for the previous invasion period of the shady slope from the first group, is E1_T. The mixed ratio of bamboo stand on the bottom, middle, and top slopes in different slope directions are listed in Table 1. A large 20 × 20 m sample square was set on each slope and five bamboo plants were selected from each quadrate using a five-point sampling method. Litter on the surface was removed, and fine roots (< 2 mm) were dug up with a spade. The rhizosphere soil was obtained by shaking off the fine roots, and then the soil within the quadrate was mixed evenly and kept at 4°C (Wang et al., 2018; Yang et al., 2019). All rhizosphere soil samples were divided into two parts, one was used for DNA extraction and the other was sieved (2 mm) for soil property analyses (Wang et al., 2017; Liu et al., 2018).

**Table 1:** Differences in bamboo standing degree of bottom slope, middle slope and top slope in different slope directions.

Item	Shady slope			Sunny slope		
	B	M	T	B	M	T
Mixed ratio (Moso bamboo:Masson pine)	22.8:1	1.31:1	0.28:1	5.56:1	0.69:1	0.28:1
Stand density of moso bamboo (tree/hm^2^)	1140±140	1175±135	640±70	1390±140	995±335	810±500

B, bottom slope; M, middle slope; T, top slope.

### 2.2. Analyses of soil properties

Soil organic matter (OM) content was calculated by multiplying soil organic carbon (SOC) content by the conventional Van Bemmelen factor of 1.724 (Lundmark et al., 2017; Yang et al., 2019). TP was determined following a complete sulfuric/perchloric acid digestion of unfiltered samples, and pH was determined in a soil–water slurry (ratio 1:2.5) with a combination pH electrode (Chen et al., 2012). The available potassium (AK), total potassium (TK), calcium (Ca), and magnesium (Mg) in the soil were determined by the atomic absorption method (Williams et al., 1966). Boron (B), hydrolyzed nitrogen (HN), available phosphorus (AP), and TN were determined by inductively coupled plasma mass spectrometry, acid hydrolysis, 0.05 mol/l HCL-0.025 mol/l H_2_SO_4_ analysis, and the semi-micro-Kjeldahl method (Xu et al., 2015), respectively.

### 2.3. Analysis of soil microorganisms

#### 2.3.1. DNA extraction

DNA extraction and sequencing were conducted by the Sangon Biotech Corporation (Shang hai, China) according to the established protocols. Total community genomic DNA extraction was performed using an E.Z.N.A. Soil DNA Kit (M5635-02, Omega, United States) following the manufacturer’s instructions. We measured the concentration of DNA using a Qubit 4.0 (Thermo Fisher, USA) to ensure that adequate amounts of high-quality genomic DNA were extracted.

#### 2.3.2. Library preparation for sequencing

The total amount of DNA input for sample preparation was 500 mg. Sequencing libraries were generated using a Hieff NGS® MaxUp II DNA Library Prep Kit for Illumina® (12200ES96, YEASEN, China) following the manufacturer’s instructions, and index codes were added to attribute sequences to each sample. Briefly, DNA was broken into fragments of about 500 bp using Covaris 220. The 500 bp library fragments were purified with Hieff NGS™ DNA Selection Beads DNA (12601ES56; YEASEN, China). The purified DNA was end-repaired and, adapter-ligated, followed by fragment selection. PCR of adapter-ligated DNA was then performed with 2 × Super Canace®II High-Fidelity Mix and Primer Mix (p5/p7). Finally, PCR products were purified (Hieff NGS™ DNA Selection Beads) and the library quality was assessed on the Qubit®4.0 Flurometer. The libraries were then quantified and pooled. Paired-end sequencing of the library was performed on the NovaSeq 6,000 sequencer (Illumina, United States). All sequence data were deposited on the NCBI and accessible *via* BioProject ID of PRJNA902136.

#### 2.3.3. Data assessment and quality control

Fastp (version 0.36) (Chen et al., 2018) was used for evaluating the quality of sequenced data. Four steps were used to filter raw reads: (1) adaptor sequences were removed; (2) low-quality bases (*Q* < 20) were removed from 3 ′ to 5 ′reads using a sliding window method (window size 4 bp); (3) pairs of overlapping reads were identified and inconsistent bases were corrected within the interval; (4) reads shorter than 35 nt and their pairing reads were removed. The remaining clean data was used for further analysis.

#### 2.3.4. Metagenome assembly and binning

First, multi-sample mixed splicing was conducted using Megahit (version 1.2.9) (Li et al., 2015) to obtain preliminary spliced contig sequences. Then, bowite2 (version 2.1.0) was used to clean the reads and, map them back to the spliced results; unmapped reads were extracted and spliced again using SPAdes (version 3.13) to obtain low-abundance contigs. MetaWRAP (version 1.3.2) was used for a series of binning, and processes, such as bin sorting, bin purification, bin quantification, bin reassembly, and bin identification, were performed in sequence. After filtering, a draft genome of a single bacteria with high integrity and low contamination was obtained.

#### 2.3.5. Gene prediction and non-redundant gene set construction

Prodigal (version 2.60) was used to predict the open reading frames (ORFs) (Rombel et al., 2022) of the splicing results, select genes longer than or equal to 100 bp, and translate them into amino acid sequences. For the gene prediction results of each sample, the CD-HIT (version 2.60) was used to reduce sequence de-redundancy and obtain a non-redundant gene set. Salmon (version 1.5.0) was used to construct a specific index of non-redundant gene sets and quantify gene abundance in each sample based on gene length by combing a dual-phase algorithm and a bias model.

#### 2.3.6. Species and functional annotations

Species and functional annotation of the genes was conducted by searching against NR,^1^ KEGG,^2^ eggnog,^3^ ARDB,^4^ CAZy,^5^ and SEED^6^ using DIAMOND (version 0.8.20), and other databases to obtain species annotation information and functional annotation information of genes. The *E*-value of 60 was used for screening. Based on gene set abundance information and annotation information, species abundance and functional abundance were obtained, and multi-directional statistical analyses, such as species and functional composition analysis, species and functional difference analysis, and sample comparison analysis were performed.

### 2.4. Statistical analysis

Path analysis was used to measure the direct and indirect effects of slope direction and position. In the path analysis, a structural equation model (SEM) is designed to measure variables and analyze the relationships among these variables in a path diagram (Fanin and Bertrand, 2016). Here, we hypothesized that microbial diversity is indirectly affected by changes in soil properties, which in turn are caused by slope direction and position (Li et al., 2017). The adequacy of the model was assessed using the *χ*^2^ tests (*p* > 0.05) and the root mean square error of approximation (RMSEA) (*p* < 0.05). These statistical tests were performed in R (version 4.2.1) using the “lavaan” package.

All data analyses were performed in R (version 4.2.1). For normally distributed data, *t*-tests were used to compare differences between two groups and one-way ANOVA to compare the differences among multiple groups. For non-normally distributed data, Wilcoxon test was used to compare the differences between two groups and Kruskal–Wallis test to compare the differences among multiple groups. Pearson’s correlation coefficient and Spearman’s rank correlations were used to determine the correlation between two normally and non-normally distributed variables, respectively. The Mantel test was performed to discern correlations of soil properties with microbial community composition, abundance, and diversity using the R (version 4.2.1) package “vegan” (Jiang et al., 2022b). Significant difference in all tests was considered at *p* < 0.05. We used Observed, Chao1, and Shannon indexes to represent composition, abundance, and diversity of microorganism, respectively. The SEM test was used to discern correlations among soil properties with microbial Observed, Chao1, and Shannon indexes using the R (version 4.2.1) package “Vegan” (Jiang et al., 2022b).

The collinear diagram, Venn diagram, and correlation heatmap were created using the R packages “circlize,” “ggplot2,” “VennDiagram,” and “corrplot,” respectively. The linear regression, redundancy analysis (RDA), and significant difference test were drawn using the R package “Vegan.”

## 3. Results

### 3.1. Variations of the microbial communities at different invasion stages

**Figure 2:**
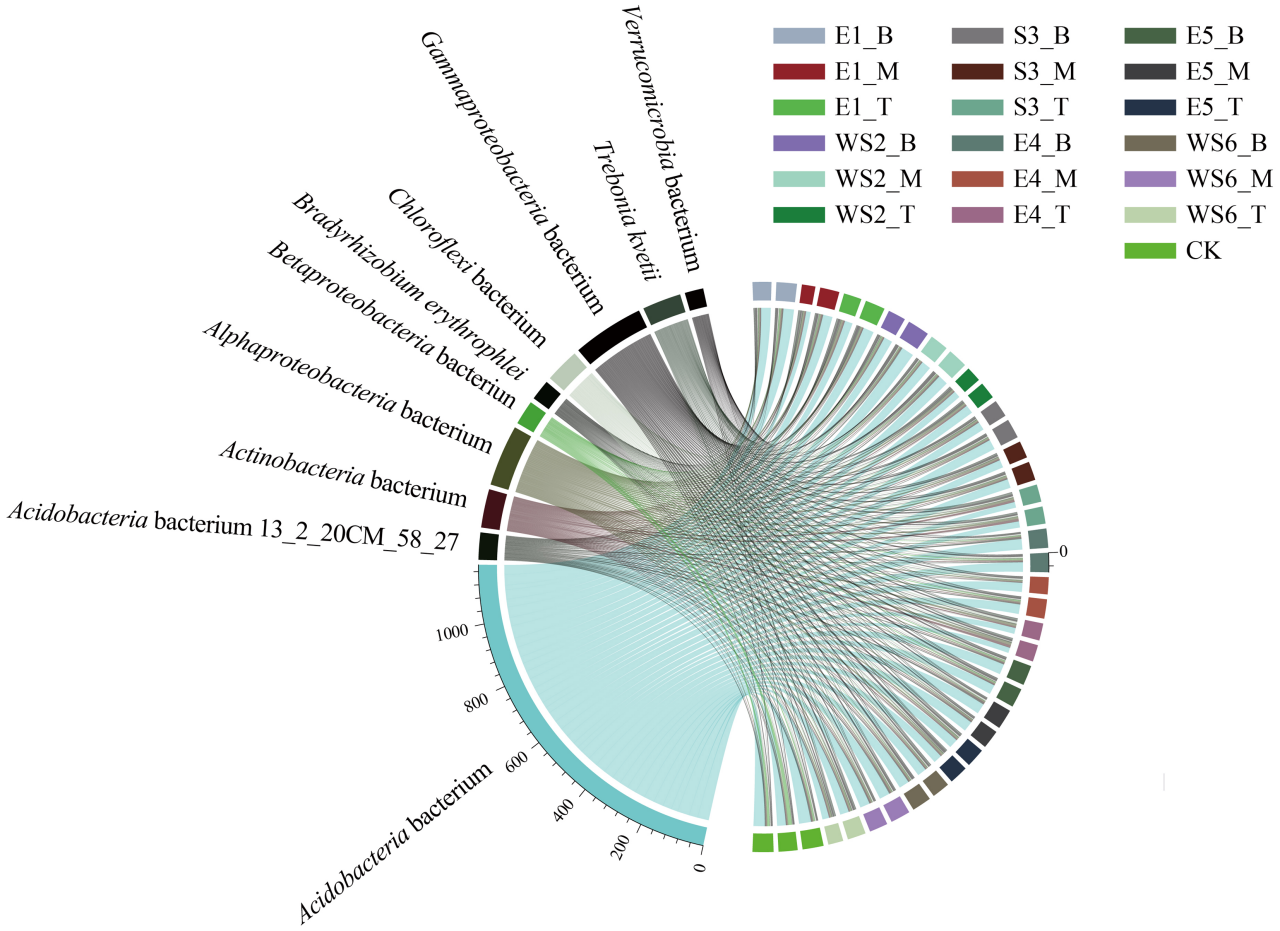
Collinear diagram of the top ten dominant microbial species from all samples.

The top 10 dominant species from all samples are shown in Figure 2, including *Acidobacteria* bacterium (53.51%), *Gammaproteobacteria* bacterium (9.88%), *Alphaproteobacteria* bacterium (8.40%), *Actinobacteria* bacterium (5.32%), *Trebonia kvetii* (5.30%), *Chloroflexi* bacterium (4.87%), *Acidobacteria* bacterium 13_2_20CM_58_27 (3.83%), *Betaproteobacteria* bacterium (3.45%), *Bradyrhizobium erythrophlei* (2.84%), and *Verrucomicrobia* bacterium (2.60%).

The variation in relative abundance of the most abundant (top 10) microbial species from the soil samples along the slope position is shown in Table 2. *Acidobacteria* bacterium and *Acidobacteria* bacterium 13_2_20CM_58_27, and *Verrucomicrobia* bacterium, had a significant difference at the bottom and top slopes (*p* < 0.05), and decreased with an increase in slope position. However, *Alphaproteobacteria* bacterium, *Actinobacteria* bacterium, *Trebonia kvetii*, and *Bradyrhizobium erythrophlei* had a significant difference at the bottom and top slopes (*p* < 0.05), which increased with an increase in slope position. However, the influence of the slope aspect on soil microbial community was not significant (*p* > 0.05) (Table 3).

**Table 2:** The variation in relative abundance of the most abundant (top ten) microbial species in samples along the slope position.

	Aci (%)	Gam (%)	Alp (%)	Bet (%)	Aci.27 (%)	Act (%)	Chl (%)	Tre (%)	Ver (%)	Bra (%)
B	33.21 ± 3.72a	6.21 ± 1.29a	3.99 ± 0.69bc	2.11 ± 0.97b	2.76 ± 1.07a	2.34 ± 0.40b	2.91 ± 0.86a	2.21 ± 0.93b	1.78 ± 0.37b	1.40 ± 0.49bc
M	30.75 ± 5.41ab	5.58 ± 1.40a	4.41 ± 0.87b	1.32 ± 0.82b	2.79 ± 1.41a	2.55 ± 0.48b	2.97 ± 1.10a	2.82 ± 1.27b	1.50 ± 0.28b	1.59 ± 0.59ab
T	25.47 ± 5.53b	5.77 ± 1.64a	6.46 ± 1.03a	0.83 ± 0.36b	0.60 ± 0.47b	4.47 ± 2.00a	2.23 ± 1.88a	4.69 ± 0.96a	0.92 ± 0.27c	2.18 ± 0.74a
CK	35.87 ± 5.85a	3.21 ± 0.27b	3.40 ± 0.51c	7.95 ± 3.94a	3.25 ± 1.20a	2.03 ± 0.38b	2.86 ± 1.46a	0.80 ± 0.05c	2.25 ± 0.30a	0.73 ± 0.14c

Data are given as mean ± SD. B, bottom slope; M, middle slope; T, top slope; Aci, *Acidobacteria* bacterium; Gam, *Gammaproteobacteria* bacterium; Alp, *Alphaproteobacteria* bacterium; Bet, *Betaproteobacteria* bacterium; Aci.27, *Acidobacteria* bacterium 13_2_20CM_58_27; Act, *Actinobacteria* bacterium; Chl, *Chloroflexi* bacterium; Tre, *Trebonia kvetii*; Ver, *Verrucomicrobia* bacterium; Bra; *Bradyrhizobium erythrophlei*. Values in the columns followed by the same letter(s) are not significantly different (*p* < 0.05) according to one-way ANOVA.

**Table 3:** The variation in relative abundance of the most abundant (top ten) microbial species in samples along the slope direction.

	Aci (%)	Gam (%)	Alp (%)	Bet (%)	Aci.27 (%)	Act (%)	Chl (%)	Tre (%)	Ver (%)	Bra (%)
Shady slope	30.03 ± 30.03a	5.94 ± 1.44a	4.92 ± 1.60a	1.64 ± 1.14b	2.29 ± 1.64a	3.00 ± 1.45a	2.91 ± 1.58a	3.13±1.50a	1.41 ± 0.43b	1.71 ± 0.72a
Sunny slope	29.59 ± 29.59a	5.76 ± 1.46a	5.00 ± 1.18a	1.20 ± 0.56b	1.81 ± 1.26a	3.24 ± 1.63a	2.50 ± 1.09a	3.35 ± 1.5a	1.39 ± 0.52b	1.73 ± 0.68a
CK	35.87 ± 5.85a	3.21 ± 0.27b	3.40 ± 0.51b	7.95 ± 3.94a	3.25 ± 1.20a	2.03 ± 0.38a	2.86 ± 1.46a	0.80±0.05b	2.25 ± 0.30a	0.73 ± 0.14b

Data are given as mean ± SD. Aci, *Acidobacteria* bacterium; Gam, *Gammaproteobacteria* bacterium; Alp, *Alphaproteobacteria* bacterium; Bet, *Betaproteobacteria* bacterium; Aci.27, *Acidobacteria* bacterium 13_2_20CM_58_27; Act, *Actinobacteria* bacterium; Chl, *Chloroflexi* bacterium; Tre, *Trebonia kvetii*; Ver, *Verrucomicrobia* bacterium; Bra, *Bradyrhizobium erythrophlei*. Values in the columns followed by the same letter(s) are not significantly different (*p* < 0.05) according to one-way ANOVA.

**Figure 3:**
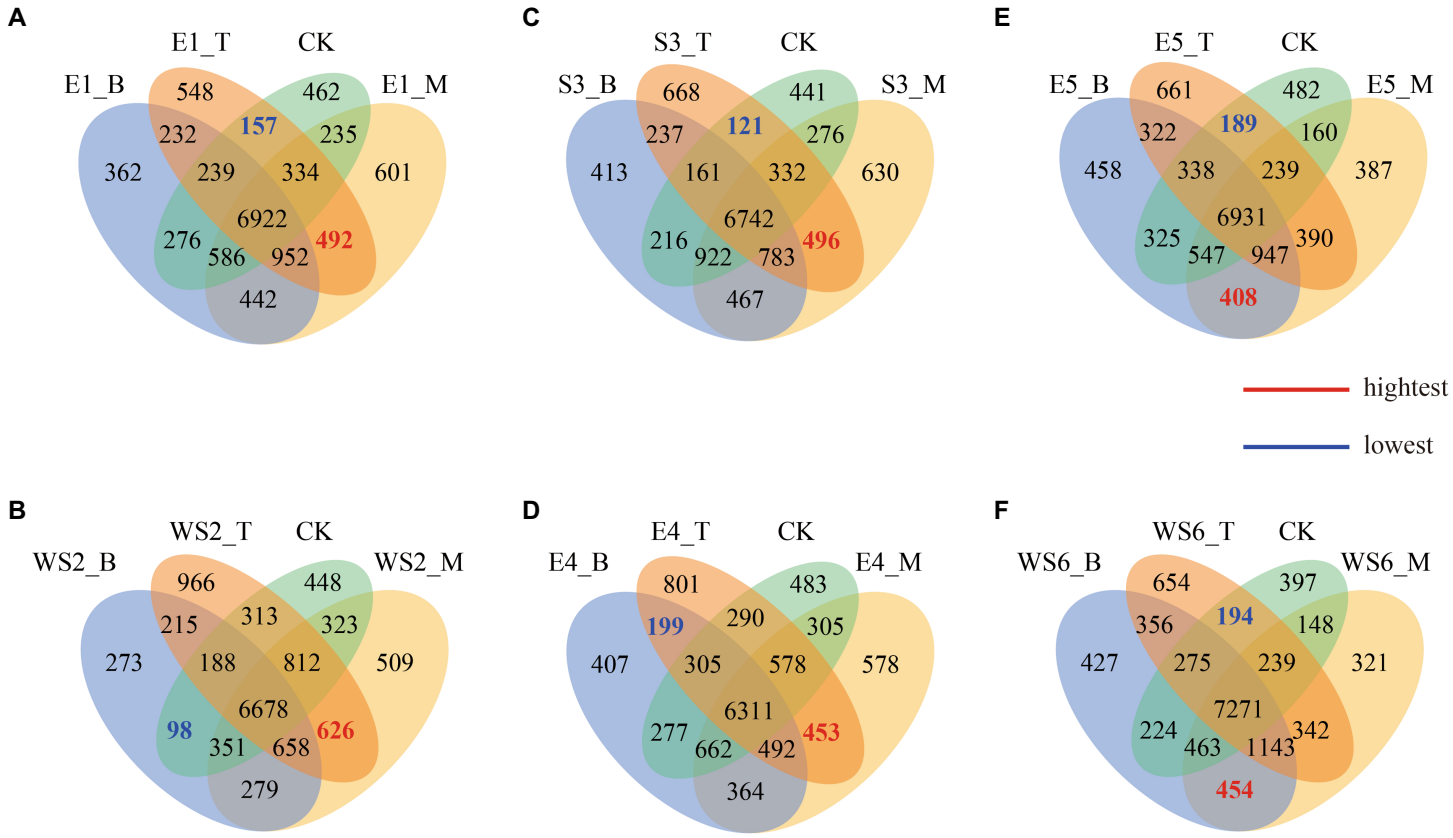
Venn diagram showing exclusive and shared OTUs of different slope positions.

Venn diagrams show the number of unique or common OTUs among different slope positions (Figure 3). In terms of the number of different OTUs, E1_B (362) < CK (462) < E1_T (548) < E1_M (601), with the highest degree of overlap between E1_ M and E1_T (492) and the lowest between E1_T and CK (157) (Figure 3A). WS2_B (273) < CK (448) < WS2_M (509) < WS2_T (966), with the highest degree of overlap between WS2_M and WS2_T (626) and the lowest between WS2_B and CK (98) (Figure 3B). S3_B (413) < CK (441) < S3_M (630) < S3_T (668), with the highest degree of overlap between S3_M and S3_T (496) and the lowest between S3_T and CK (121) (Figure 3C). E4_B (407) < CK (483) < E4_M (578) < E4_T (801), with the highest degree of overlap between E4_M and E4_T (453) and the lowest between E4_B and E4_T (199) (Figure 3D). E5_M (387) < E5_B (458) < CK (482) < E5_T (661), with the highest degree of overlap between E5_B and E5_M (408) and the lowest between E5_T and CK (189) (Figure 3E). WS6_M (321) < CK (397) < WS6_B (427) < WS6_T (654), with the highest degree of overlap between WS6_B and WS6_M (454) and the lowest between WS6_T and CK (194) (Figure 3F). Our results show that the overlap between the middle slope and the top or bottom slopes is higher than that of the top and bottom slopes.

### 3.2. Relationships between soil properties and microbial communities

We compared the soil properties of shady and sunny slopes (Table 4) and the results showed that the Mg content was significantly different in slope direction (*p* < 0.05), however other soil properties had no significant difference (*p* > 0.05). Table 5 indicates the difference in soil properties at different slope positions of the shady slope. The pH was significantly different at the bottom and top slopes (*p* < 0.05), and it decreased with the increase in slope position. The OM, AP, and Ca content was also significantly different at the bottom and top slopes (*p* < 0.05), however it increased with the increase in slope position. The TN content was not significantly different at the bottom and top slopes (*p* > 0.05), but it increased with the increase in slope position. The different soil properties at different slope positions of the sunny slope are shown in Table 6. The results indicated that OM, AP, Ca, TK, Mg, and HN were significantly different at the bottom and top slopes (*p* < 0.05), and increased with the increase in slope position. The TN content was not significantly different at the bottom and top slopes (*p* > 0.05), but it increased with the increase in slope position.

**Table 4:** The difference of soil properties in different slope directions.

	Shady slope	Sunny slope	CK
pH	4.79 ± 0.24b	4.80 ± 0.25b	5.60 ± 0.17a
OM (g/kg)	6.12 ± 2.74a	5.95 ± 3.02a	4.00 ± 1.69a
AP (mg/kg)	1.13 ± 1.13a	1.10 ± 1.1a	0.57 ± 0.57a
B (mg/kg)	94.97 ± 94.97a	90.68 ± 90.68a	78.50 ± 78.5b
Ca (g/kg)	0.88 ± 0.88b	0.86 ± 0.86b	1.29 ± 1.29a
TK (g/kg)	21.40 ± 21.4a	18.82 ± 18.82a	10.73 ± 10.73b
Mg (g/kg)	4.44 ± 0.34a	4.01 ± 0.48b	2.54 ± 0.19c
TN (g/kg)	2.07 ± 0.61a	2.01 ± 0.62a	2.21 ± 0.34a
TP (g/kg)	0.35 ± 0.05b	0.33 ± 0.06b	0.47 ± 0.02a
HN (mg/kg)	180.92 ± 45.84a	175.19 ± 48.52a	194.67 ± 21.39a
AK (mg/kg)	79.18 ± 18.28a	69.45 ± 13.23a	65.57 ± 14.72a

Data are given as mean ± SD. Values in the columns followed by the same letter(s) are not significantly different (*p* < 0.05) according to one-way ANOVA.

**Table 5:** The difference of soil properties in different slope positions of shady slope.

	B	M	T	CK
pH	4.90 ± 0.2b	4.83 ± 0.21bc	4.64 ± 0.25c	5.60 ± 0.17a
OM (g/kg)	4.17 ± 1.12b	5.39 ± 1.18b	8.79 ± 2.95a	4.00 ± 1.69b
AP (mg/kg)	0.85 ± 0.33b	0.91 ± 0.35b	1.64 ± 0.87a	0.57 ± 0.2b
B (mg/kg)	98.40 ± 7.75a	96.33 ± 10.23a	90.19 ± 9.81a	78.50 ± 3.4b
Ca (g/kg)	0.70 ± 0.18c	0.85 ± 0.29bc	1.09 ± 0.26ab	1.29 ± 0.44a
TK (g/kg)	21.53 ± 1.59a	21.62 ± 1.87a	21.05 ± 3.83a	10.73 ± 0.49b
Mg (g/kg)	4.38 ± 0.25a	4.59 ± 0.14a	4.35 ± 0.5a	2.54 ± 0.19b
TN (g/kg)	1.74 ± 0.32b	1.98 ± 0.32ab	2.48 ± 0.81ab	2.21 ± 0.3a
TP (g/kg)	0.34 ± 0.05b	0.33 ± 0.02b	0.37 ± 0.07b	0.47 ± 0.02a
HN (mg/kg)	164.00 ± 38a	169.00 ± 29a	210.00 ± 54a	195.00 ± 21a
AK (mg/kg)	85.01 ± 18.54a	82.14 ± 18.96a	70.39 ± 15.12a	60.57 ± 14.72a

Data are given as mean ± SD. B, bottom slope; M, middle slope; T, top slope. Values in the columns followed by the same letter(s) are not significantly different (*p* < 0.05) according to one-way ANOVA.

**Table 6:** The difference of soil properties in different slope positions of sunny slope.

	B	M	T	CK
pH	4.88 ± 0.18b	4.87 ± 0.25b	4.64 ± 0.25b	5.60 ± 0.17a
OM (g/kg)	3.86 ± 0.89b	5.18 ± 2.29b	8.79 ± 2.95a	4.00 ± 1.69b
AP (mg/kg)	0.80 ± 0.47b	0.86 ± 0.4b	1.64 ± 0.87a	0.57 ± 0.2b
B (mg/kg)	86.92 ± 6.78a	94.93 ± 6.24a	90.19 ± 9.81a	78.50 ± 3.4b
Ca (g/kg)	0.69 ± 0.17b	0.82 ± 0.14b	1.09 ± 0.26a	1.29 ± 0.44a
TK (g/kg)	16.45 ± 1.77b	18.97 ± 1.07ab	21.05 ± 3.83a	10.73 ± 0.49c
Mg (g/kg)	3.68 ± 0.35b	4.01 ± 0.35ab	4.35 ± 0.5a	2.54 ± 0.19c
TN (g/kg)	1.73 ± 0.28b	1.83 ± 0.35b	2.48 ± 0.81ab	2.21 ± 0.34a
TP (g/kg)	0.30 ± 0.05b	0.32 ± 0.05b	0.37 ± 0.07b	0.47 ± 0.02a
HN (mg/kg)	157.50 ± 34.43b	158.08 ± 36.94b	210.00 ± 54.39a	194.67 ± 21.39ab
AK (mg/kg)	66.13 ± 15.03a	71.84 ± 9.12a	70.39 ± 15.12a	65.57 ± 14.72a

Data are given as mean ± SD. B, bottom slope; M, middle slope; T, top slope. Values in the columns followed by the same letter(s) are not significantly different (*p* < 0.05) according to one-way ANOVA.

**Figure 4:**
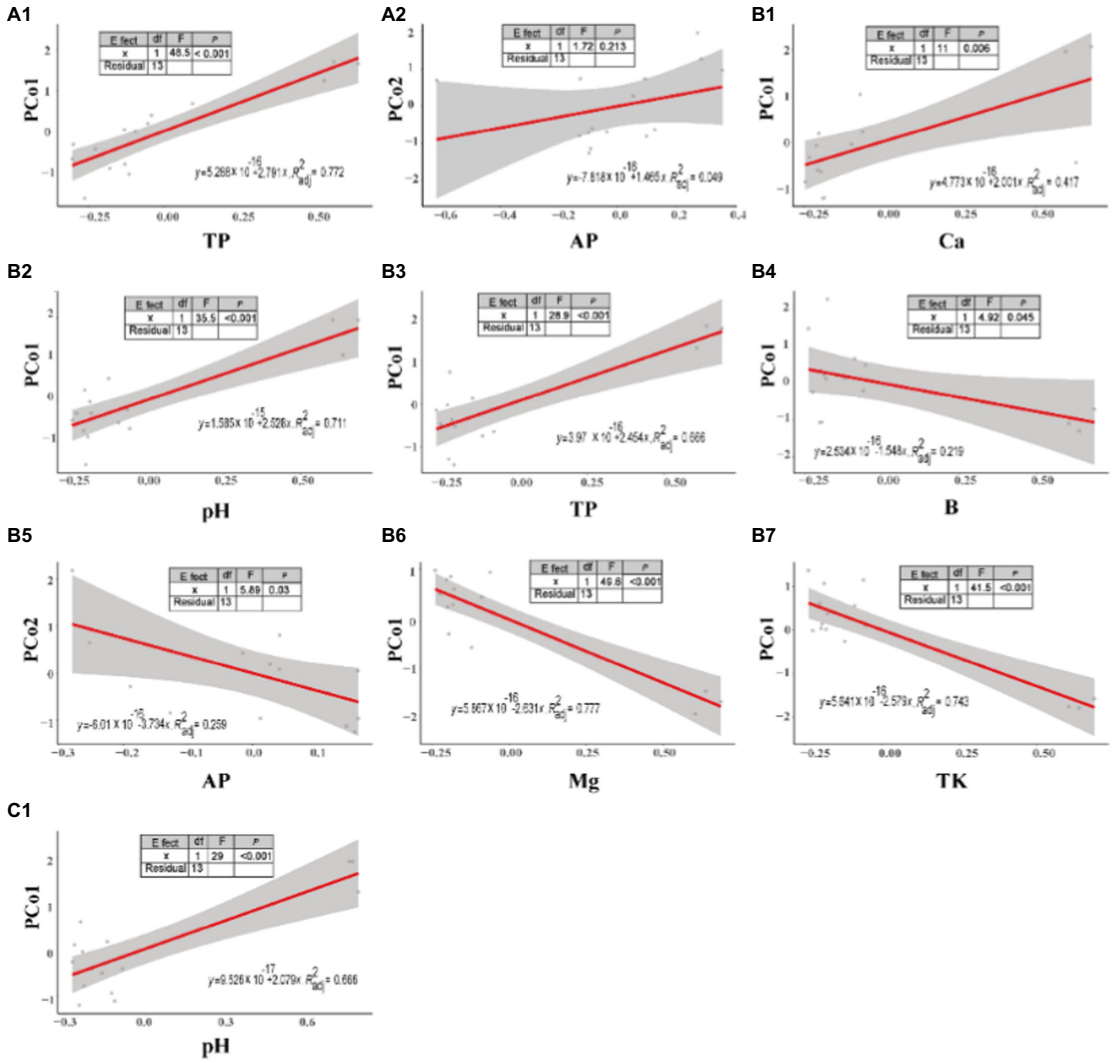
Linear regression between soil properties and microbial abundance at different slope positions. **(A)** bottom, **(B)** middle, and **(C)** top slopes.

Linear regression indicated the relationship between microbial abundance and soil properties (Figure 4). On the bottom slopes of the mountain (Figure 4A), microbial abundance showed a significant positive correlation with soil TP (*p <* 0.001, *F =* 48.5), but an insignificant positive correlation with AP (*p =* 0.213, *F =* 1.72). On the middle slopes of the mountain (Figure 4B), microbial diversity was positively correlated with soil Ca (*p =* 0.006, *F =* 11), pH (*p <* 0.001, *F =* 35.5) and TP (*p <* 0.001, *F =* 28.9), but negatively correlated with soil B (*p =* 0.045, *F =* 4.92), AP (*p =* 0.03, *F =* 5.89), Mg (*p <* 0.001, *F =* 49.6), and TK (*p <* 0.001, *F =* 41.5). On the top slopes of the mountain (Figure 4C), soil microbial abundance was significant positively correlated with pH (*p <* 0.001, *F =* 29). However, the correlation between microbial abundance and other soil properties was not linear.

**Figure 5:**
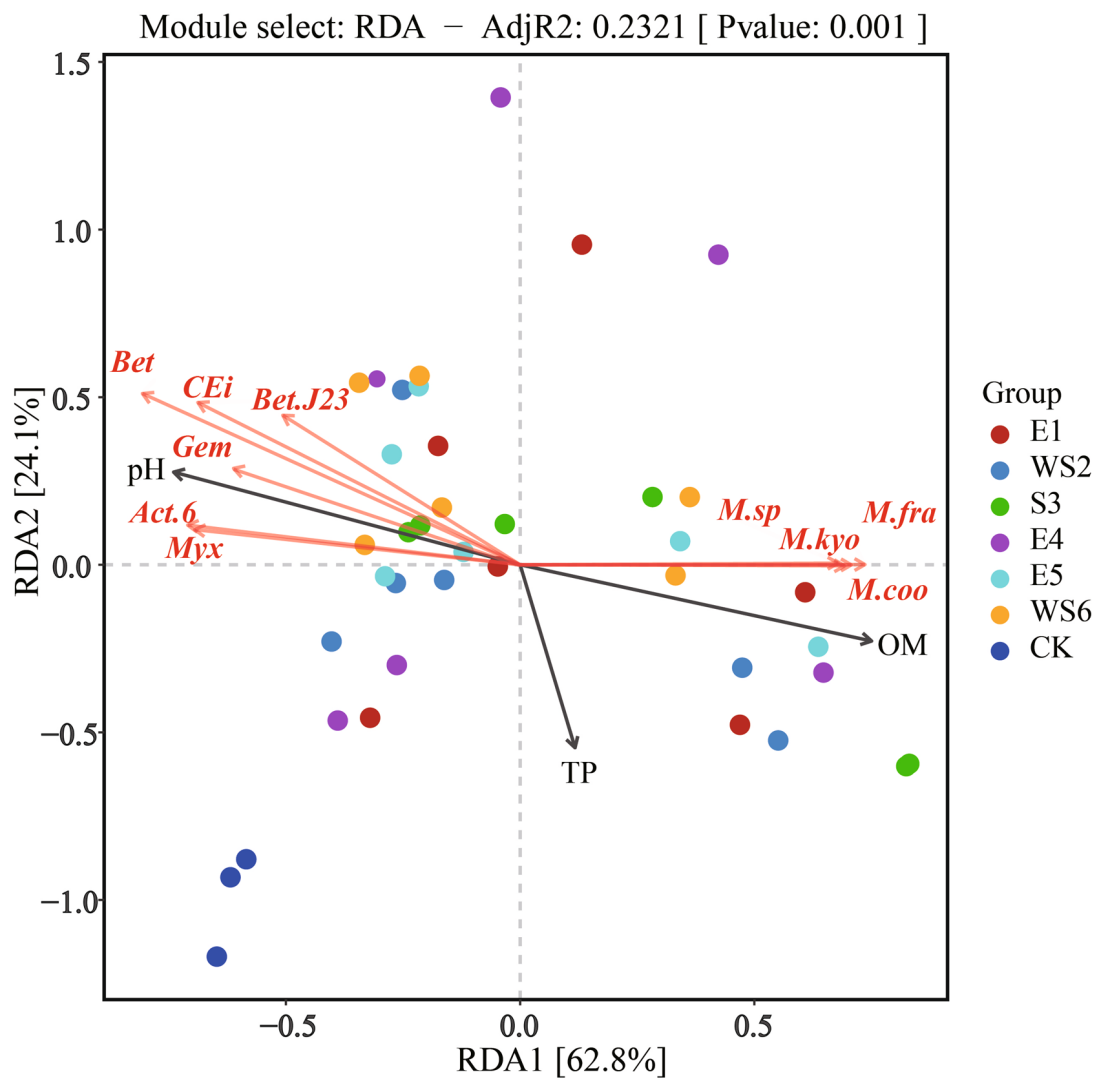
RDA analysis between microbial community and soil properties. *Bet*, *Betaproteobacteria* bacterium; *CEi*, *Candidatus Eisenbacteria* bacterium; *Bet.J23*, *Betaproteobacteria* bacterium SCGC AG−212−J23; *Gem*, *Gemmatimonadetes* bacterium; *Act.6*, *Actinobacteria* bacterium 13_2_20CM_2_66_6; *Myx*, *Myxococcaceae* bacterium; *M.sp*, *Mycobacterium* sp. 1245111.1; *M.fra*, *Mycobacterium fragae*; *M.kyo*, *Mycobacterium kyorinense*; *M.coo*, *Mycobacterium cookii*.

The RDA of soil properties and the top 1% of the microbial community under every group was chosen to further investigate the relationship between environmental variables and microbial communities. The results are shown in Figure 5. Strong evidence from the first axis of the RDA two-dimensional diagram was found to explain 62.8% of the data, and the second axis accounted for 24.1%. *Betaproteobacteria* bacterium, *Candidatus Eisenbacteria* bacterium, *Betaproteobacteria* bacterium SCGC_AG-212-J23, *Gemmatimonadetes* bacterium, *Actinobacteria* bacterium 13_2_20CM_2_66_6 and *Myxococcaceae* bacterium correlated positively with pH, but negatively with OM and TP. However, *Mycobacterium* sp. 1245111.1, *Mycobacterium fragae, Mycobacterium kyorinense and Mycobacterium cookii* correlated positively with OM and TP, but negatively with pH.

**Figure 6:**
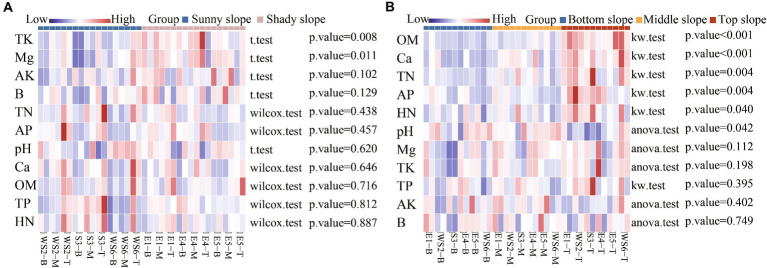
Correlation heatmap between soil properties and slope direction **(A)** and position **(B)**.

In order to distinguish the effects of slope direction and position on soil properties, a significant difference analysis was performed. Correlation heat maps showed significant differences in the effects of slope direction on TK (*p =* 0.008) and Mg (*p =* 0.011), and that TK and Mg were higher on shady slopes and lower on sunny slopes (Figure 6A). Significant differences were found for the effects of slope position on OM (*p <* 0.001), Ca (*p <* 0.001), TN (*p =* 0.004), AP (*p =* 0.004), HN (*p =* 0.040), and pH (*p =* 0.042). OM, Ca, TN, AP, HN content was higher on the top slope than on the bottom and middle slopes. pH was higher on the middle slope than on the bottom slope (Figure 6B).

### 3.3. Slope direction and position influence soil microbial communities

**Figure 7:**
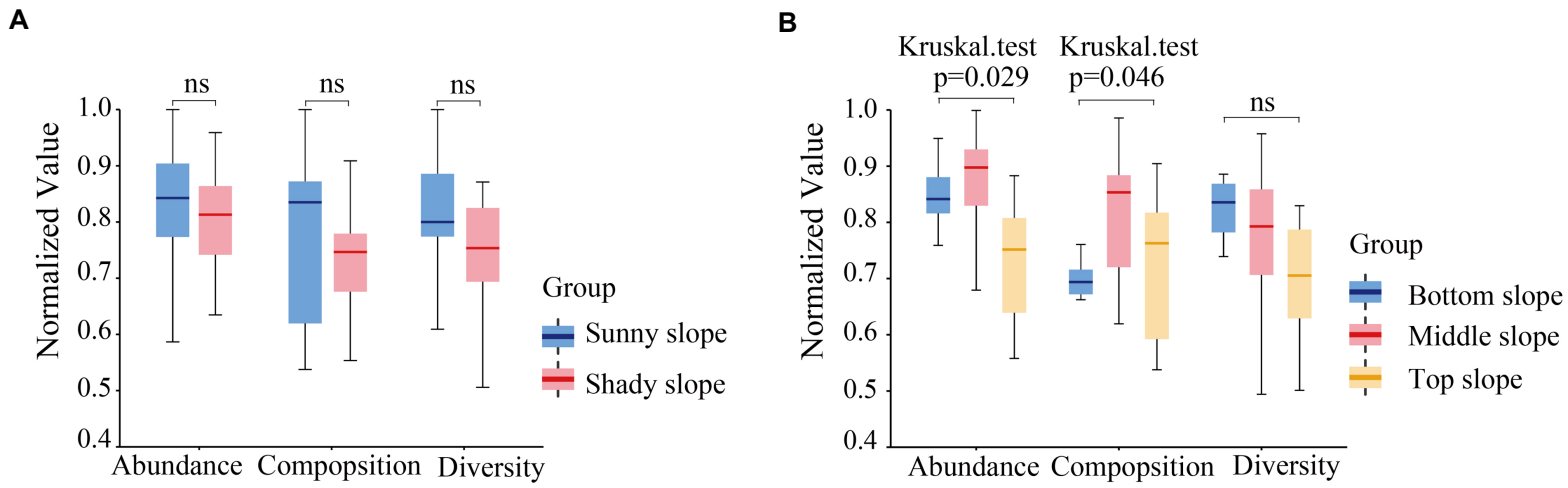
Significant difference of microbial abundance, composition, and diversity on slope direction **(A)** and position **(B)**.

In order to analyze the influence of slope direction and position on soil microbial communities, we first analyzed the significance of their influence on the three soil microbial indices. The box-plot of sample distance indicated that the differences between the effect of slope direction on microbial abundance, composition, and diversity were not significant (Figure 7A). However, the difference was significant between the effect of slope position on microbial abundance (*p =* 0.029) and composition (*p =* 0.046), but not on microbial diversity (Figure 7B).

**Figure 8:**
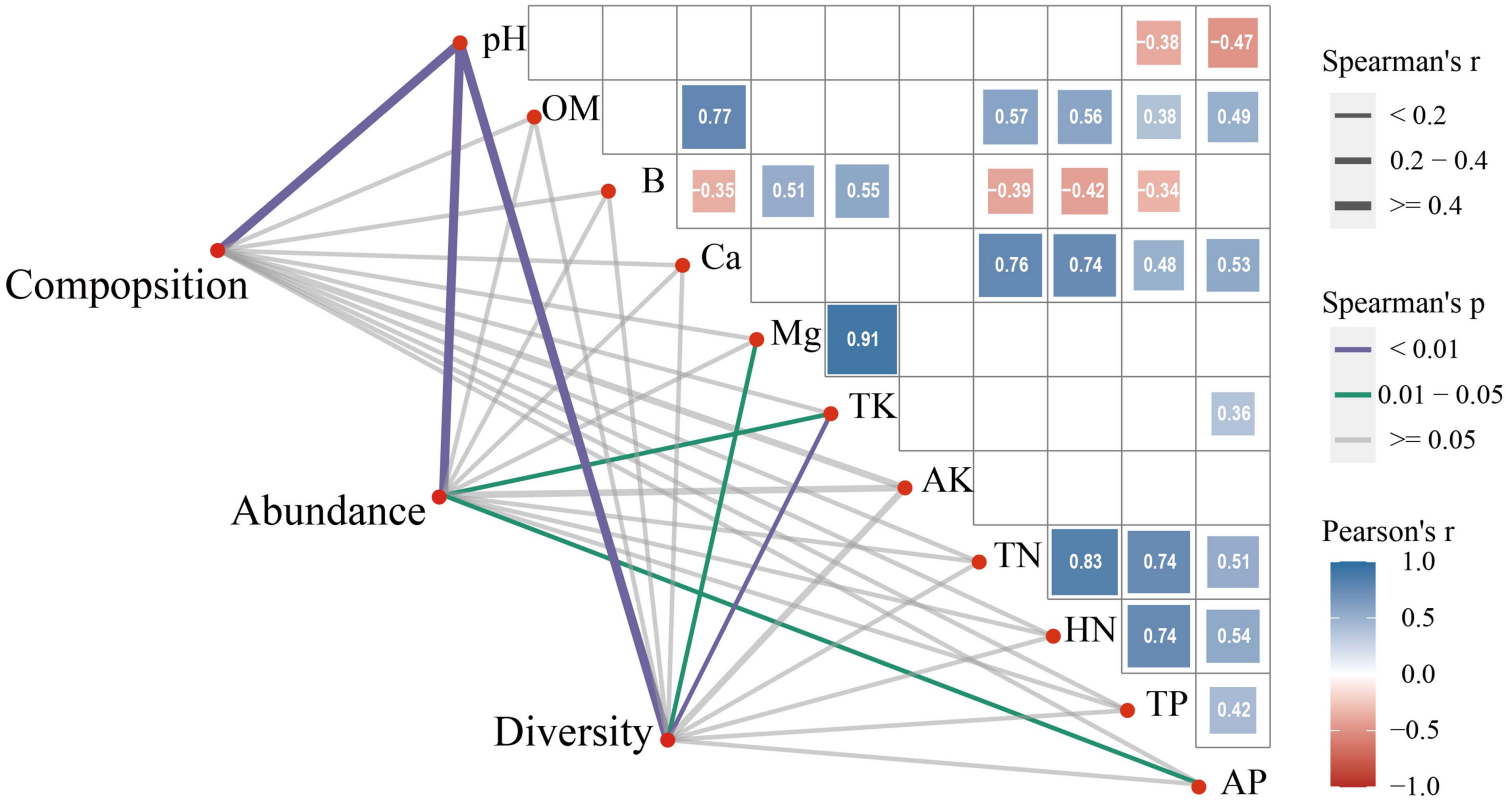
Correlation of soil properties with microbial composition, abundance, and diversity.

To further understand the relationship between soil microbial indices and soil properties, we used a correlation analysis. The results showed that microbial composition was strongly and significantly correlated with pH (*r =* 0.445, *p* = 0.003), while abundance was significantly and strongly correlated with soil pH (*r =* 0.563, *p* = 0.002), TK (*r =* −0.1148, *p* = 0.043), and AP (*r =* −0.484, *p* = 0.049). Soil microbial diversity had a significant and strong correlation with pH (*r =* 0.394, *p* = 0.003) and a significant but weak correlation with Mg (*r =* −0.284, *p* = 0.050) and TK (*r =* −0.253, *p* = 0.010) (Figure 8).

The correlation analysis of soil properties showed that pH was negatively correlated with TP (*r =* −0.38, *p* = 0.024) and AP (*r =* −0.47, *p* = 0.004). OM was positively correlated with Ca (*r =* 0.77, *p* < 0.001), TN (*r =* 0.57, *p* < 0.001), HN (*r =* 0.56, *p* < 0.001), TP (*r =* 0.38, *p* = 0.023), and AP (*r =* 0.49, *p* = 0.002). B was negatively correlated with Ca (*r =* −0.35, *p* = 0.036), TN (*r =* −0.39, *p* = 0.018), HN (*r =* −0.42, *p* = 0.011), and TP (*r =* −0.34, *p* = 0.046), but positively correlated with Mg (*r =* 0.51, *p* = 0.001) and TK (*r =* 0.55, *p* = 0.001). Ca was positively correlated with TN (*r =* 0.76, *p* < 0.001), HN (*r =* 0.74, *p* < 0.001), TP (*r =* 0.48, *p* = 0.003), and AP (*r =* 0.53, *p* = 0.001). Mg was positively correlated with TK (*r =* 0.91, *p* < 0.001). TK was positively correlated with AP (*r =* 0.36, *p* = 0.030). TN was positively correlated with HN (*r =* 0.83, *p* < 0.001), TP (*r =* 0.74, *p* < 0.001), and AP (*r =* 0.51, *p* = 0.001). HN was positively correlated with TP (*r =* 0.74, *p* < 0.001) and AP (*r =* 0.54, *p* = 0.001). TP was positively correlated with AP (*r =* 0.42, *p* = 0.011) (Figure 8).

**Figure 9:**
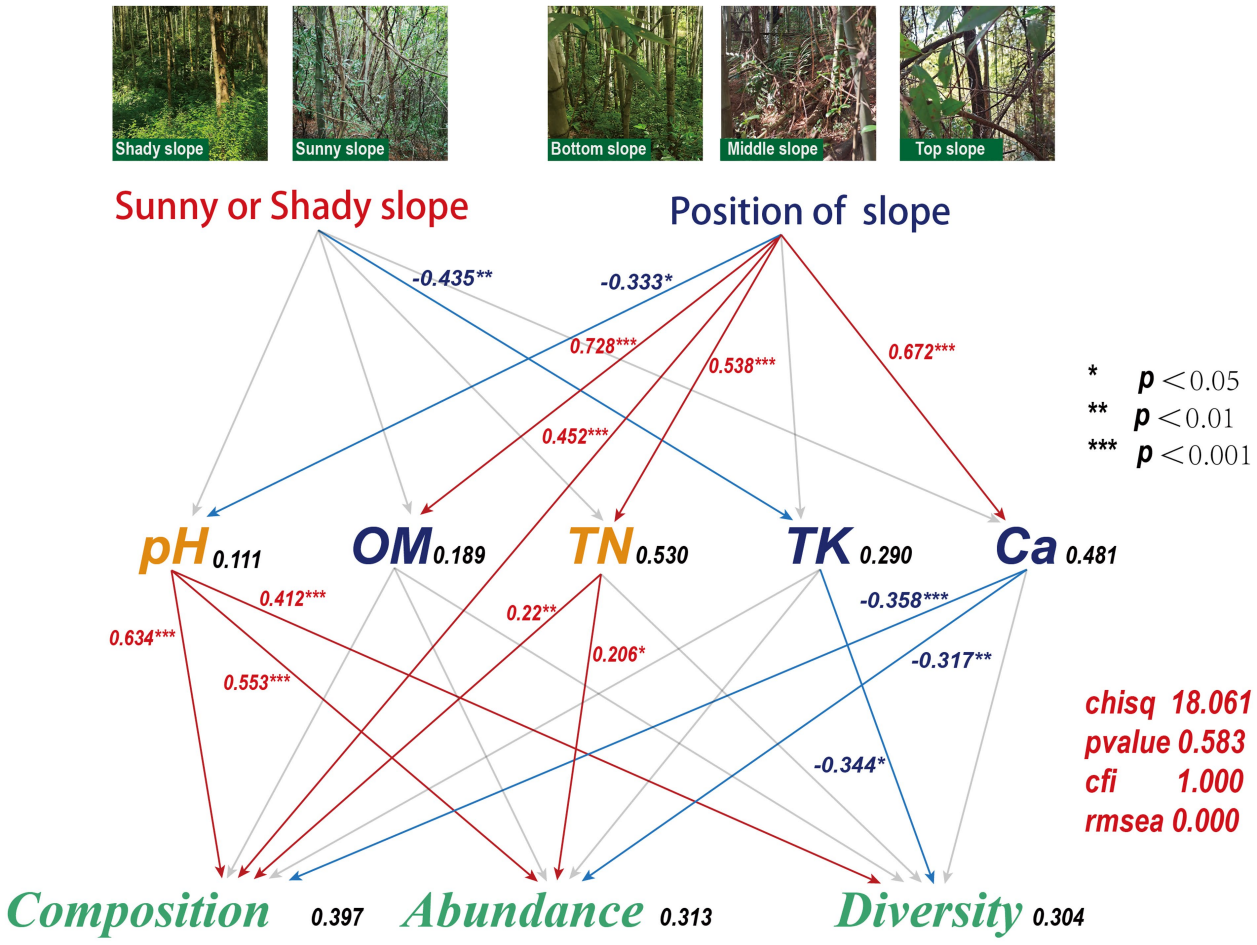
A structural equation model for the relationship between soil properties and microbial community composition, abundance, and diversity as influenced by slope direction and slope position. The number in the right of each of the soil or microbial parameter is the squared-multiple correlation, and the number on each line between these parameters is the standardized regression-weight (*: significant at *p* < 0.05; **: significant at *p* < 0.01; ***: significant at *p* < 0.001). Red solid lines indicate that the standardized regression weights are negative, blue solid lines indicate that the standardized regression weights are positive, and gray dashed lines indicate that the standardized regression weights are not significant (*p* ≥ 0.05) and thus their statistics are not shown.

We hypothesized that changes in soil properties caused by the direction and position of the slope would have different effects on microbial composition, abundance, and diversity. We tested our hypothesis using SEM analyses. The direction and position of the slope directly induced changes in soil properties (pH: 11.1%, OM: 18.9%, TN: 53.0%, TK: 29.0%, Ca: 48.1%). Sunny or shady slopes had a significant negative relationship with TK (*r =* −0.435, *p* = 0.004) content. Slope position had a significant negative relationship with pH (*r =* −0.333, *p* = 0.034), but a positive relationship with OM (*r =* 0.728, *p* < 0.001), TN (*r =* 0.538, *p* < 0.001), and Ca (*r =* 0.672, *p* < 0.001) content. pH was significantly positively related to composition (*r =* 0.634, *p* < 0.001), abundance (*r =* 0.553, *p* < 0.001), and diversity (*r =* 0.412, *p* = 0.002). The TN content was significantly positively related to composition (*r =* 0.220, *p* = 0.014) and abundance (*r =* 0.206, *p* = 0.013), the TK content was negatively related to diversity (*r =* −0.344, *p* = 0.011), the Ca content was negatively related to composition (*r =* −0.358, *p* = 0.003) and abundance (*r =* −0.317, *p* = 0.003), and slope position significantly positively affected composition (*r =* 0.452, *p* < 0.001) (Figure 9).

## 4. Discussion

### 4.1. Response of soil microbial communities variation to moso bamboo invasion

When comparing the relative abundance of the top 10 microbial species at different slope positions (Table 2), the present study found that the abundance of *Acidobacteria* bacterium and *Acidobacteria* bacterium 13_2_20CM_58_27, and *Verrucomicrobia* bacterium was significantly different between the top and the bottom slopes (*p* < 0.05), the relative abundance at the top slope was low, while the relative abundance at the bottom slope was high. The abundance of *Alphaproteobacteria* bacterium, *Actinobacteria* bacterium, *Trebonia kvetii*, and *Bradyrhizobium erythrophlei* was also significantly different between the top and bottom slopes (*p* < 0.05), but their relative abundance was higher on the top slope. *Acidobacteria* bacterium and *Acidobacteria* bacterium 13_2_20CM_58_27 belong to the *Acidobacteria* phylum. A previous study, which reported *Acidobacteria* as the dominant flora in karst areas (Jiang et al., 2022c), which are widely distributed and have specific ecological functions in forest soil (Liu et al., 2017). *Acidobacteria* are involved in nitrogen fixation, organic matter decomposition, and plant growth promotion and they are sensitive to soil pH, soil temperature, and plant diversity (Zhang et al., 2014; Kim et al., 2021). Our study found that the relative abundance of *Acidobacteria* was the highest in all samples, which is consistent with other studies (Liu et al., 2017; Jiang et al., 2022c). Although the pH of the bottom slope was higher than that of the top slope, both had low pH, which is suitable for the growth of *Acidobacteria*. The relative abundance of *Acidobacteria* on the bottom slope was higher because of *Acidobacteria* are oligotrophs and versatile heterotrophs (Lin et al., 2013), better adapted to extreme environments. *Alphaproteobacteria* bacterium and *Bradyrhizobium erythrophlei* belong to the *Proteobacteria* phylum. *Proteobacteria* include species attributed to nitrogen fixation, organic matter decomposition, and plant growth promotion (Zhang and Xu, 2008; Yarwood et al., 2009). *Alphaproteobacteria* bacterium and *Bradyrhizobium erythrophlei* were higher at the top slope, possibly due to the high OM content in the top slope. *Actinobacteria bacterium* and *Trebonia kvetii* belong to the *Actinobacteria* phylum. *Actinobacteria* are ubiquitous in the soil owing to their potential to grow in extreme environments by deploying a defense system that stems from their ability to produce secondary metabolites (Lee et al., 2018). *Actinobacteria* produce extracellular hydrolases that decompose the biomass of animals or plants, rendering them the central organisms in the carbon cycle. For example, decomposition of different organic substances such as cellulose, organic acids and humus is possible *via* the production of cellulase and chitinase (El-Tarabily et al., 1996; Ranjani et al., 2016; Bhatti et al., 2017). In our study, *Actinobacteria* bacterium and *Trebonia kvetii* had higher relative abundance in the top slope. This may be due to the nutrient cycling system of the original Masson pine forest being disturbed during the invasion of moso bamboo. *Actinobacteria* bacterium and *Trebonia kvetii* failed to adapt to new conditions, which affected their survival and decreased their relative abundance in the soil. *Verrucomicrobia* bacterium belong to the *Verrucomicrobia* phylum. Shen et al. (2017) indicated that *Verrucomicrobia* had a strong correlation with pH. However, other soil features, such as C:N ratio, soil moisture, TC, and TN, also significantly affected the *Verrucomicrobia*. In our study, *Verrucomicrobia* bacterium also showed higher abundance in the bottom slope, possibly relative to soil pH.

The Venn diagram shows that the top slope had a higher overlap with the middle slope than with the bottom slope (Figure 3), indicating that moso bamboo invasion changes the OTUs of the microbial community, probably due to the changes in the original vegetation community caused by the invasion. These changes cause differences in underground root secretions and surface apoplast, which impact the soil carbon source and nutrient cycling, leading to changes in the microbial community (Millard and Singh, 2010). Invasion of broadleaf evergreen forests by moso bamboo reduces the active organic carbon and nitrogen content of the soil, decreases the nutrient content of the original soil, and changes the structure of the original soil organic carbon and nitrogen pool (Zhang et al., 2019).

### 4.2. Key soil properties closely related to microbial communities

By comparing the soil properties of different slope directions, we found that Mg content showed significant differences (*p* < 0.05); and the Mg content in the shady slope was higher than that in the sunny slope (Table 4). By comparing the soil properties of different slope positions, we found that pH was significantly different on the slope position of the shady slope (*p* < 0.05), and decreased with the increase in slope position, but not significantly different on the slope position of the sunny slope (*p* > 0.05). The OM, AP, and Ca content was significantly different at the bottom and the top slope (*p* < 0.05), and increased with the increase of slope position in both shady and sunny slopes (Tables 5, 6). RDA analysis showed that pH, TP, and OM were the key factors that influenced the microbial communities (Figure 5). This suggests that microbial abundance in the previous invasive and invasive stages of the moso bamboo invasion is positively correlated with pH. Similar results have been observed in other case studies. Numerous studies (Zhang et al., 2017; Liu et al., 2021) have shown that soil microbial communities are highly sensitive to changes in pH. Invasion of moso bamboo forests has led to changes in pH and microbial diversity, and there is strong evidence that pH is a major controlling factor of the composition of soil bacterial and fungal communities (Fierer and Jackson, 2006; Nicol et al., 2008). For example, Li et al. (2022) reported that the increase in bacterial abundance can be attributed to the increased pH in the bamboo-invaded site, because pH tends to increase bacterial diversity. Zhao et al. (2019) found that soil pH and microbial community structure are indirectly driven by plants. Plant root exudates and organic acids produced during litter decomposition were the main factors that decreased the soil pH. In addition, hot and humid climate conditions and the higher concentration of calcium in karst mountain areas promote the growth of microorganisms and decrease soil acid reactions, which affects soil microbial composition and diversity. Our study found that microbial abundance was positively correlated with TP on the bottom and middle slopes. Other studies have reported similar results, indicating that among the soil properties, TN, TP, and pH have the greatest influence on soil bacterial diversity (Guo et al., 2020). In addition, Jiang et al. (2022a) found that soil microbial communities were more limited by C and P in the karst ecosystem of Tiankeng.

### 4.3. Influence mechanisms of slope direction and position on soil microbial communities

The structural equation model values (chisq = 18.061, *value of p* = 0.583, cfi = 1.000, rmsea = 0) confirm the goodness of the model fit and the feasibility of the simulation results. Although soil microorganisms also affected soil properties, our goal was to explore the mechanism by which slope direction and slope position affected microorganisms. The results showed that slope direction indirectly affects microbial diversity through TK (Figure 9). Similar results were obtained by Bai et al. (2016), who showed that slope direction has a significant effect on the composition, structure, and function of microbial communities in karst sink holes. Soil TK plays an important role in the accumulation of biomass and the restoration of forest vegetation in karst areas, and the TK content decreases during the accumulation of biomass and the enrichment of plant species (Shen et al., 2020). Similar negative correlation between TK and microbial diversity was found in our study. Other studies have reported that slope-induced factors, such as TP, TN, TK, pH, and soil water content are important sources of ectomycorrhizal fungal richness (Wei et al., 2021). It has also been found that slope direction influences nitrogen morphology and the proportion of each nitrogen state, which differs from our findings and may be caused by differences in the study area (Huang et al., 2015). Since slope direction is a factor that indirectly affects TK variation, the analysis suggests that factors such as light and moisture may also influence microbial diversity. Differences in microbial diversity on shaded and sunny slopes may be due to differences in the microclimate created by slope direction (Chu et al., 2016).

The top, middle, and bottom slopes represent the previous invasion, invasion, and late invasion stages of moso bamboo, respectively. In general, our results showed that slope position had a significant effect on microbial composition and microbial abundance, but not on microbial diversity (Figure 7). Slope position was directly negatively correlated with pH, and positively correlated with OM, TN, and *Ca.* pH was positively correlated with microbial composition, abundance, and diversity; TN was positively correlated with microbial composition and abundance, and Ca was negatively correlated with microbial composition and abundance (Figure 9). The result indicated a positive correlation between pH and microbial diversity, which is similar to previous findings by Wan et al. (2021), who considered that spatial differences in soil microbial diversity are caused by pH-driven organic phosphorus mineralization. Previous studies (Ouyang et al., 2022) have also confirmed the increase in forest soil pH caused by bamboo invasion. Soil TN is a key soil environmental factor affecting microbial diversity. In this study, we found a positive correlation between TN and microbial composition and abundance, thus corroborating previous studies. For example, soil TN has the greatest effect on soil microbial diversity on the Loess Plateau, and greatly affects fungal community structure (Guo et al., 2020). Li et al. (2021) also found a significant positive correlation between TN and bacterial populations in their study on the Tibetan Plateau. Karst soils are characterized by a lack of nitrogen and phosphorus, but high Ca and Mg content (Li et al., 2019).The positive effect of slope position on Ca may indicate that moso bamboo exacts different limitations on soil Ca at different stages of invasion, which in turn may indirectly affect soil microbial diversity. Previous studies (Li et al., 2013) have revealed that moso bamboo affects soil nutrients and carbon inputs, which are important factors influencing the structure of the soil microbial community. A quantitative study on the variation in pH, Ca, and TN by slope position and the variation in TK by slope direction identified the key soil properties that affect the changes in microbial composition, abundance and diversity caused by moso bamboo invasion. The differences between our study and previous studies may be due to the unique nature of the high Ca and Mg content in karst soils (Li et al., 2019).

## 5. Conclusion

This study provides a comprehensive assessment of the composition, abundance and diversity of microorganisms during bamboo invasion in the Lijiang River Basin. Our results showed that the abundance of *Acidobacteria* bacterium, *Acidobacteria* bacterium 13_2_20CM_58_27, and *Verrucomicrobia* bacterium decreased, while that of *Alphaproteobacteria* bacterium, *Actinobacteria* bacterium, *Trebonia kvetii*, and *Bradyrhizobium erythrophlei* increased as the slope increased (*p* < 0.05). However, the difference of slope direction on microbial community was not significant. Most soil microorganisms were significantly positively correlated with soil pH and were significantly negatively correlated with soil OM and TP. The abundance and composition of microbial communities significantly differed with slope position. Slope position indirectly affects microbial composition, abundance, and diversity through pH, OM, TN, and Ca; however, it can directly influence microbial composition. Slope direction indirectly affects microbial diversity through TK. This study elucidates the response mechanisms of soil microbial composition, abundance, and diversity to moso bamboo invasion, and these results can help with the development of management strategies for the Lijiang River Basin.

## Data availability statement

The datasets presented in this study can be found in online repositories. The names of the repository/repositories and accession number(s) can be found below: https://www.ncbi.nlm.nih.gov/, PRJNA902136.

## Author contributions

HY and JM: original draft preparation and methodology. HS and WH: performed experiments, analyzed data, and wrote manuscript. YD, LA, MW, JH, ZZ, and ML: conducted field sampling and plot surveys. All authors contributed to the article and approved the submitted version.

## Funding

This research was funded by the Basic Ability Enhancement Program for Young and Middle-aged Teachers of Guangxi (2021KY0058), Guangxi Key Research and Development Projects (Guike AB21220057), National Natural Science Foundation (32260387), and Key R & D Projects in Guangxi (Guike AB22080071).

## Conflict of interest

The authors declare that the research was conducted in the absence of any commercial or financial relationships that could be construed as a potential conflict of interest.

## Publisher’s note

All claims expressed in this article are solely those of the authors and do not necessarily represent those of their affiliated organizations, or those of the publisher, the editors and the reviewers. Any product that may be evaluated in this article, or claim that may be made by its manufacturer, is not guaranteed or endorsed by the publisher.

## Footnotes

^1^ http://ncbi.nlm.nih.gov/

^2^ http://www.kegg.jp

^3^ http://eggnogdb.embl.de/

^4^ https://ardb.cbcb.umd.edu/

^5^ http://www.cazy.org/

^6^ http://www.theseed.org/wiki/Main_Page
