# Supplementary material for: Moso bamboo (Phyllostachys edulis (Carrière) J. Houzeau) invasion affects soil microbial communities in adjacent planted forests in the Lijiang River basin, China
Source: Front Microbiol. 2023 Feb 21;14:1111498. doi: 10.3389/fmicb.2023.1111498 (PMC9990415; doi:10.3389/fmicb.2023.1111498)
Supplement: Supplementary file 4 [file Data_Sheet_2.PDF]

```
lavaan 0.6-12 ended normally after 47 iterations

  Estimator                                         ML
  Optimization method                           NLMINB
  Number of model parameters                        32

  Number of observations                            36

Model Test User Model:
                                              Standard      Robust
  Test Statistic                                18.060      16.822
  Degrees of freedom                                20          20
  P-value (Chi-square)                           0.583       0.665
  Scaling correction factor                                  1.074
    Satorra-Bentler correction

Model Test Baseline Model:

  Test statistic                               333.291     242.154
  Degrees of freedom                                44          44
  P-value                                        0.000       0.000
  Scaling correction factor                                  1.376

User Model versus Baseline Model:

  Comparative Fit Index (CFI)                    1.000       1.000
  Tucker-Lewis Index (TLI)                       1.015       1.035

  Robust Comparative Fit Index (CFI)                         1.000
  Robust Tucker-Lewis Index (TLI)                            1.028

Loglikelihood and Information Criteria:

  Loglikelihood user model (H0)               -246.982    -246.982
  Loglikelihood unrestricted model (H1)       -237.952    -237.952

  Akaike (AIC)                                 557.964     557.964
  Bayesian (BIC)                               608.636     608.636
  Sample-size adjusted Bayesian (BIC)          508.669     508.669

Root Mean Square Error of Approximation:

  RMSEA                                          0.000       0.000
  90 Percent confidence interval - lower         0.000       0.000
  90 Percent confidence interval - upper         0.128       0.114
  P-value RMSEA <= 0.05                          0.682       0.762
```

```
  Robust RMSEA                                                0.000
  90 Percent confidence interval - lower                      0.000
  90 Percent confidence interval - upper                      0.122

Standardized Root Mean Square Residual:

  SRMR                                           0.097        0.097

Parameter Estimates:

  Standard errors                             Standard
  Information                                 Expected
  Information saturated (h1) model          Structured

Regressions:
                   Estimate  Std.Err  z-value  P(>|z|)   Std.lv  Std.all
  Observed ~
    pH                0.634    0.130    4.872    0.000    0.634    0.655
    OM               -0.022    0.059   -0.373    0.709   -0.022   -0.023
    TN                0.220    0.090    2.453    0.014    0.220    0.227
    Ca               -0.358    0.119   -3.023    0.003   -0.358   -0.359
    TK               -0.239    0.125   -1.907    0.057   -0.239   -0.247
position            0.452    0.093    4.840    0.000    0.452    0.467
  Chao1 ~
    pH                0.553    0.139    3.968    0.000    0.553    0.563
    TN                0.206    0.083    2.476    0.013    0.206    0.209
    Ca               -0.317    0.106   -2.987    0.003   -0.317   -0.313
    TK               -0.235    0.136   -1.731    0.083   -0.235   -0.239
position              0.156    0.085    1.828    0.068    0.156    0.159
  Shannon ~
    pH                0.412    0.135    3.049    0.002    0.412    0.424
    TK               -0.344    0.135   -2.542    0.011   -0.344   -0.353
  pH ~
position             -0.333    0.157   -2.117    0.034   -0.333   -0.333
  TK ~
direction           -0.435    0.150   -2.897    0.004   -0.435   -0.435
  OM ~
  position              0.728    0.114    6.369    0.000    0.728    0.728
  TN ~
 position             0.538    0.140    3.831    0.000    0.538    0.538
  Ca ~
position              0.672    0.116    5.782    0.000    0.672    0.694

Covariances:
                   Estimate  Std.Err  z-value  P(>|z|)   Std.lv  Std.all
 .OM ~~
   .Ca                0.164    0.063    2.597    0.009    0.164    0.353
```

```
  .TN ~~
    .Ca               0.338    0.101    3.360    0.001    0.338    0.592
  .pH ~~
    .Ca               0.210    0.085    2.477    0.013    0.210    0.328
  .Observed ~~
    .Chao1            0.570    0.137    4.151    0.000    0.570    0.958
    .Shannon          0.526    0.132    3.984    0.000    0.526    0.888
  .Chao1 ~~
    .Shannon          0.587    0.145    4.052    0.000    0.587    0.916

Variances:
                   Estimate  Std.Err  z-value  P(>|z|)   Std.lv  Std.all
    .Observed         0.550    0.130    4.243    0.000    0.550    0.603
    .Chao1            0.643    0.152    4.243    0.000    0.643    0.687
    .Shannon          0.639    0.151    4.243    0.000    0.639    0.696
    .pH               0.865    0.204    4.243    0.000    0.865    0.889
    .TK               0.788    0.186    4.243    0.000    0.788    0.811
    .OM               0.457    0.108    4.243    0.000    0.457    0.470
    .TN               0.691    0.163    4.243    0.000    0.691    0.710
    .Ca               0.473    0.100    4.714    0.000    0.473    0.519

R-Square:
                   Estimate
    Observed          0.397
    Chao1             0.313
    Shannon           0.304
    pH                0.111
    TK                0.189
    OM                0.530
    TN                0.290
    Ca                0.481


  chisq      df  pvalue     cfi   rmsea     bic    srmr
 18.060  20.000   0.583   1.000   0.000 608.636   0.097
```
